# Methylmercury, an environmental electrophile capable of activation and disruption of the Akt/CREB/Bcl-2 signal transduction pathway in SH-SY5Y cells

**DOI:** 10.1038/srep28944

**Published:** 2016-06-30

**Authors:** Takamitsu Unoki, Yumi Abiko, Takashi Toyama, Takashi Uehara, Koji Tsuboi, Motohiro Nishida, Toshiyuki Kaji, Yoshito Kumagai

**Affiliations:** 1 Faculty of Medicine, University of Tsukuba, Tsukuba, Ibaraki 305-8575, Japan; 2 Department of Medicinal Pharmacology, Graduate School of Medicine, Dentistry, and Pharmaceutical Sciences, Okayama University, Okayama 700-8530, Japan; 3 Division of Cardiocirculatory Signaling, Okazaki Institute for Integrative Bioscience (National Institute for Physiological Sciences), National Institutes of Natural Sciences, Okazaki, Aichi 444-8787, Japan; 4 Department of Translational Pharmaceutical Sciences, Graduate School of Pharmaceutical Sciences, Kyushu University, Fukuoka 812-8582, Japan; 5 Faculty of Pharmaceutical Sciences, Tokyo University of Science, Noda, Chiba 275-8510, Japan

## Abstract

Methylmercury (MeHg) modifies cellular proteins via their thiol groups in a process referred to as “*S*-mercuration”, potentially resulting in modulation of the cellular signal transduction pathway. We examined whether low-dose MeHg could affect Akt signaling involved in cell survival. Exposure of human neuroblastoma SH-SY5Y cells of up to 2 μM MeHg phosphorylated Akt and its downstream signal molecule CREB, presumably due to inactivation of PTEN through *S*-mercuration. As a result, the anti-apoptotic protein Bcl-2 was up-regulated by MeHg. The activation of Akt/CREB/Bcl-2 signaling mediated by MeHg was, at least in part, linked to cellular defence because either pretreatment with wortmannin to block PI3K/Akt signaling or knockdown of Bcl-2 enhanced MeHg-mediated cytotoxicity. In contrast, increasing concentrations of MeHg disrupted Akt/CREB/Bcl-2 signaling. This phenomenon was attributed to *S*-mercuration of CREB through Cys286 rather than Akt. These results suggest that although MeHg is an apoptosis-inducing toxicant, this environmental electrophile is able to activate the cell survival signal transduction pathway at lower concentrations prior to apoptotic cell death.

Methylmercury (MeHg) exists as a naturally occurring substance or industrially produced environmental contaminant and is known to be a neurotoxicant that affects the central nervous system resulting in disorders such as Minamata disease in Japan and MeHg poisoning in Iraq^1^,^2^. MeHg-mediated toxicity is, at least in part, due to its electrophilic nature, which allows it to easily react with nucleophiles such as protein thiol groups, resulting in the disruption of cellular homeostasis through the *S*-mercuration of proteins^3^,^4^. We have reported that MeHg covalently modifies a variety of proteins such as manganese superoxide dismutase (Mn-SOD), neuronal nitric oxide synthase (nNOS), arginase I, sorbitol dehydrogenase (SDH), and ubiquitin *C*-terminal hydrolase L1 (UCH-L1), resulting in their disruption^5^,^6^,^7^,^8^,^9^. In contrast, the environmental electrophile 1,2-naphthoquinone activates protein tyrosine phosphatase (PTP) 1B/epidermal growth factor receptor (EGFR) signalling, which is involved in cell survival, and kelch-like ECH-associated protein 1 (Keap1)/nuclear factor (erythroid-derived 2)-like 2 (Nrf2) signalling, which regulates phase-II xenobiotic detoxification enzymes and phase-III transporters associated with cellular protection against this electrophile through *S*-arylation of PTP1B and Keap1^10^,^11^. These observations suggest that MeHg may also be associated with not only disruption of cellular homeostasis, but also activation of the cellular defence system at low doses through induction of electrophilic signal transduction pathways resulting from *S*-mercuration of proteins. Consistent with this notion, we also found that MeHg activates Nrf2 through *S*-mercuration of Keap1 and up-regulates its downstream proteins, strongly suggesting that MeHg may activate other cellular signal transduction pathways. These observations led us to assume that MeHg exposure would result in a biphasic response with respect to cell death at high concentrations and survival at low concentrations.

Several lines of evidence have indicated that MeHg is a toxic metal that causes apoptosis in a variety of cells such as rat pheochromocytoma PC12s^12^, primary cultured rat granule neurons^13^, and human T-cells^14^, presumably through induction of apoptotic signaling. However, Chen *et al*.^15^ previously reported that MeHg is also able to activate Akt signaling at lower concentrations, possibly through generation of reactive oxygen species, although the mechanistic details were not provided.

Akt activation is negatively regulated by phosphatase and tensin homolog deleted from chromosome 10 (PTEN)^16^. Once Akt is activated, a transcription factor cAMP response element-binding protein (CREB) undergoes phosphorylation by activated Akt, thereby up-regulating one of its downstream genes, B-cell lymphoma 2 (Bcl-2), an anti-apoptotic protein^17^,^18^,^19^. Our preliminary examination indicated that MeHg did cause Akt phosphorylation at a non-toxic concentration, whereas increasing concentrations of MeHg blocked Akt activation and resulted in concomitant apoptotic cell death in human neuroblastoma SH-SY5Y cells. Taken together, we hypothesized that activation of such different signalling pathways associated with cell survival and cell death could be dependent on the examined concentration and thus covalent modification of cellular proteins by MeHg seems to play a role in the observed phenomena. To address this issue, we explored MeHg-mediated non-linear Akt/CREB/Bcl-2 signal transduction and contribution of *S*-mercuration to modulation of the signaling.

## Results

### Activation of the Akt/CREB/BCl-2 signaling pathway by lower concentrations of MeHg in SH-SY5Y cells

**Figure 1:**
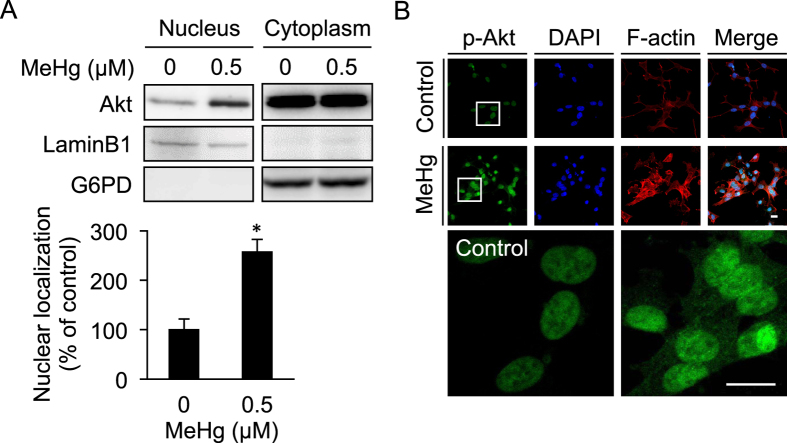
Translocation of phosphorylated Akt in the nucleus. SH-SY5Y cells were exposed to 0.5 μM of MeHg for 6 h. Nuclear and cytoplasmic fractions were subjected to Western blotting with indicated antibodies. Nuclear Akt band intensity in control was defined as 100%. *p < 0.05 *vs*. control. Each value is the mean ± S.D. of three determinations (**A**). The localization of phosphorylated-Akt (p-Akt) (green) was detected along with F-actin (red) and the nucleus (blue). The regions enclosed by the white squares are magnified in the lower panels. Scale bars, 10 μm. (**B**).

**Figure 2:**
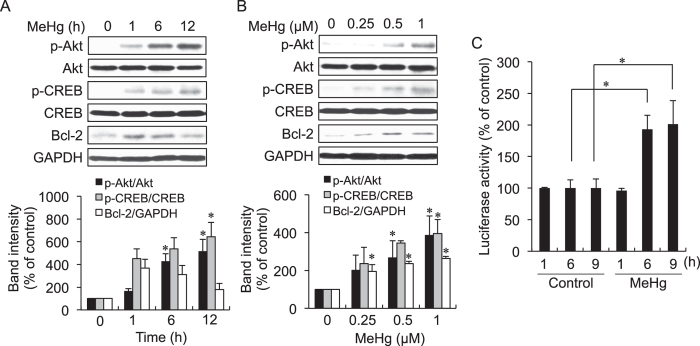
Activation of Akt and CREB and induction of Bcl-2 by MeHg in SH-SY5Y cells. The cells were exposed to 1 μM of MeHg for the indicated time and then were subjected to western blotting and the band intensities were quantified (**A**). The cells were exposed to the indicated concentrations of MeHg for 6 h. The whole lysates were subjected to western blotting and the band intensities were quantified (**B**). The cells, transfected with CRE-luciferase and the pRLTK expressed vector, were exposed to 1 μM of MeHg for the indicated time. The luciferase assay was performed as described in the materials and methods (**C**). *p < 0.05 *vs*. control. Each value is the mean ±  S.D. of three determinations.

When SH-SY5Y cells were exposed to 0.5 μM MeHg for 6 h, the phosphorylated-Akt level was enhanced and Akt was translocated to the nucleus as evaluated by Western blotting (Fig. 1A) and immuno-histochemical assay (Fig. 1B). Under these conditions, globular actin was converted into its polymerized form (F-actin, Fig. 1B), suggesting a cellular response to MeHg. As shown in Fig. 2A,B, MeHg activated Akt in a time- and concentration- (up to 1 μM) dependent manner as determined by western blot analysis. Under these conditions, CREB was also phosphorylated during exposure to MeHg (Fig. 2A,B). MeHg up-regulated Bcl-2, an anti-apoptotic protein and CREB-target gene, through transactivation of the cAMP response element (CRE) (Fig. 2C). Under this condition, Glycogen synthase kinase-3β (GSK-3β) was also phosphorylated and Nrf2 was activated by MeHg exposure (see Supplementary Fig. S1).

**Figure 3:**
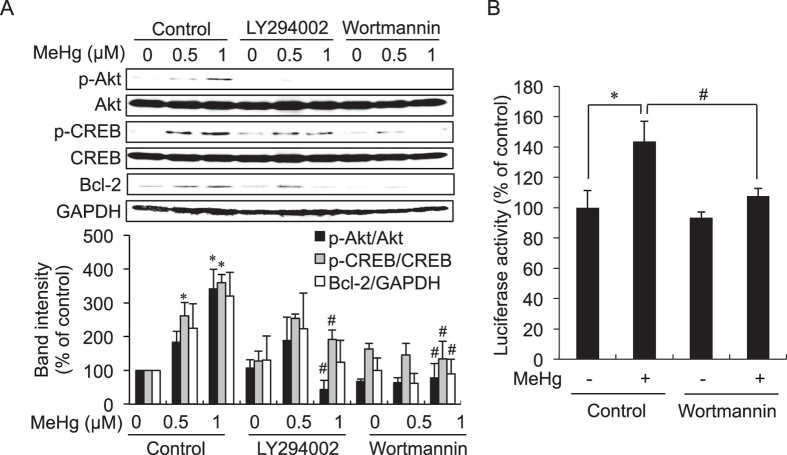
Inhibition of the MeHg-mediated Akt/CREB/Bcl-2 signaling pathway by an Akt inhibitor in SH-SY5Y cells. SH-SY5Y cells were pretreated with wortmannin (0.5 μM) for 30 min, and then the cells were exposed to MeHg for 12 h. The cells were also exposed to MeHg for 12 h in the presence or absence of LY294002 (5 μM). The whole cell lysates were subjected to western blotting and the band intensities were quantified (**A**). The cells were transfected with CRE-luciferase and then treated with wortmannin (0.5 μM) for 30 min following exposure of MeHg for 6 h. The luciferase assay was performed as described in the materials and methods (**B**). *p < 0.05 *vs*. control MeHg (0 μM). ^#^*p* < 0.05 *vs*. control MeHg (0.5 μM or 1 μM). Each value is the mean ±  S.D. of three determinations.

Pretreatment with either LY294002 or wortmannin, a specific inhibitor for phosphatidylinositol 3-kinase (PI3K), markedly inhibited MeHg-mediated phosphorylation of Akt and CREB and suppressed Bcl-2 protein expression (Fig. 3A). The MeHg-induced luciferase activity of CRE was also inhibited by wortmannin (Fig. 3B). These observations support that MeHg is able to activate the Akt/CREB/Bcl-2 signal transduction pathway at concentrations up to 1 μM.

### Contribution of the MeHg-mediated Akt/CREB/Bcl-2 signaling pathway to cytotoxicity by MeHg in SH-SY5Y cells

**Figure 4:**
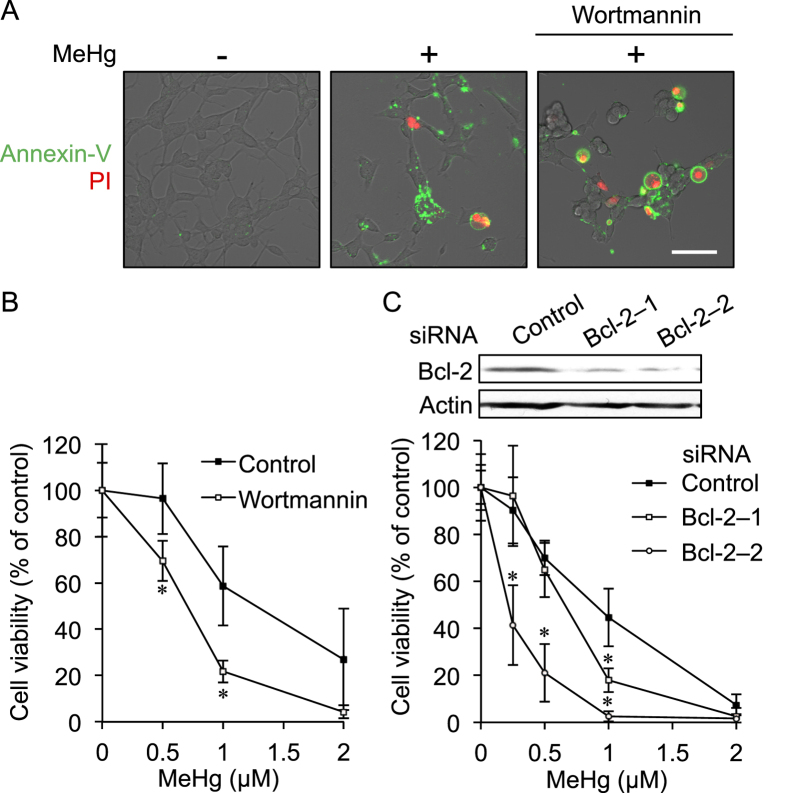
Protective role of Akt/CREB/Bcl-2 signaling in MeHg-induced cell death in SH-SY5Y cells. SH-SY5Y cells were pretreated with wortmannin (5 μM) for 30 min and then the cells were exposed to MeHg (1 μM) for 6 h. The cells were stained with annexin V and propidium iodide (PI). Scale bar, 50 μm (**A**). The cells were pretreated with wortmannin (0.5 μM) for 30 min following exposure of MeHg for 24 h, and then an MTT assay was performed (**B**). The cells were transfected with control, Bcl-2–1, or Bcl-2–2 siRNA for 48 h and the expression level of Bcl-2 was examined by western blotting (**C**, upper). The siRNA transfected cells were exposed to MeHg for 24 h, and then cell viability was measured by the MTT assay (**C**, lower). *p < 0.05 *vs*. control. Each value is the mean ±  S.D. of three determinations.

To investigate whether or not up-regulation of Bcl-2 through activation of Akt/CREB signaling mediated by MeHg at lower concentrations could participate in substantial repression of MeHg-mediated apoptotic cell damage, histochemical determination and the MTT assay were carried out in SH-SY5Y cells with and without pretreatment of wortmannin. As shown in Fig. 4A,B, wortmannin treatment increased the number of propidium iodide positive cells and significantly decreased cell viability during exposure to MeHg. Knockdown of Bcl-2 also significantly accelerated MeHg-mediated cytotoxicity. These results suggest that activating the Akt/CREB signal transduction pathway caused by treatment with MeHg certainly diminishes late apoptosis and its cytotoxicity during MeHg exposure, indicative of a hormetic response.

### Disruption of Akt/CREB/Bcl-2 signaling during exposure of SH-SY5Y cells to higher concentrations of MeHg

**Figure 5:**
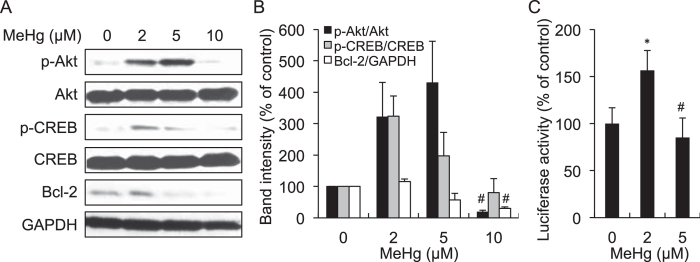
Disruption of Akt/CREB/Bcl-2 signaling by higher concentrations of MeHg in SH-SY5Y cells. SH-SY5Y cells were exposed to MeHg (0, 2, 5, and 10 μM) for 6 h, and subjected to western blotting (**A**) and the band intensities were quantified (**B**). The cells were transfected with CRE-luciferase and exposed to MeHg (0, 2, and 5 μM) for 3 h. The luciferase assay was performed as described in the materials and methods (**C**). *p < 0.05 *vs*. control MeHg (0 μM). ^#^*p* < 0.05 *vs*. control MeHg (2 μM). Each value is the mean ±  S.D. of three determinations.

As discussed herein and reported by others, there is little doubt that MeHg causes apoptotic cell death^12^,^13^,^14^. We therefore hypothesized that while MeHg can phosphorylate Akt and CREB leading to increased Bcl-2 protein at lower concentrations, this organometal may also disrupt Akt/CREB/Bcl-2 signalling at higher concentrations. As shown in Fig. 5A,B, activation of Akt mediated by MeHg reached a plateau at 5 μM and then declined at 10 μM in SH-SY5Y cells. However, while CREB and Bcl-2 were activated at 2 μM MeHg, they were markedly blocked in the presence of higher concentrations of MeHg with no further increase in phosphorylation and expression. A similar effect was also observed for treatment of MeHg on CRE-dependent luciferase activity in cells (Fig. 5C).

### *S*-Mercuration of PTEN, Akt and CREB proteins

**Figure 6:**
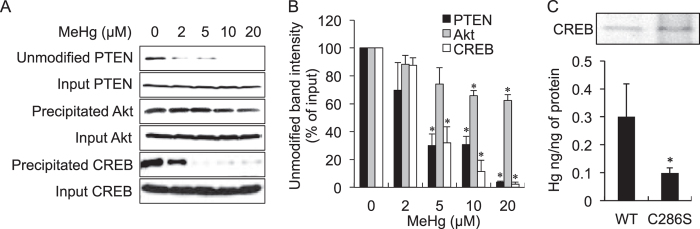
*S*-Mercuration of Akt and CREB by MeHg in SH-SY5Y cells. SH-SY5Y cells were treated with MeHg for 3 h and a BPM-precipitation assay was performed. The precipitated proteins were detected by western blotting with the indicated antibodies (**A**). The cell lysate before the separation was used to estimate the alteration of protein expression by MeHg stimulation. The band intensities were quantified (**B**). Recombinant CREB (0.5 mg/mL) was reacted with MeHg (20 μM) for 30 min at 37 °C, and then the proteins were subjected to SDS-PAGE following Coomassie brilliant blue staining (**C**, upper). The CREB protein on the gel was cut out using a gel-band cutter and the total Hg concentration in the CREB was analysed by AAS. The mercury content was expressed as Hg ng/ng of protein (**C**, lower). *p < 0.05 *vs.* control. Each value is the mean ±  S.D. of three determinations.

It is well recognized that oxidative modification and/or inhibition of PTEN is involved in the activation of Akt^20^,^21^,^22^,^23^,^24^. Because *S*-mercuration of PTEN is classified as an oxidation of thiol groups in PTEN, we speculated that MeHg-mediated activation of the Akt/CREB/Bcl-2 signalling pathway might be caused by inhibition of PTEN through *S*-mercuration and thus could be detected by modification using the biotin-PEAC_5_-maleimide (BPM)-precipitation assay^25^,^26^. As expected, a MeHg concentration-dependent decrease of BPM-labelled PTEN was observed (Fig. 6A,B) and activation of recombinant human PTEN was significantly inhibited by MeHg (see Supplementary Fig. S2), suggesting that inhibition of PTEN activity may be attributed to *S*-mercuration of the protein by MeHg.

We previously found that the atmospheric electrophile 1,2-NQ covalently modifies CREB through reaction at Cys286, resulting in a repression of its DNA binding activity through CRE, thereby down-regulating Bcl-2^25^,^27^. This finding led us to assume that because MeHg is electrophilic, it may also modify CREB. To address this issue, we performed the BPM-precipitation assay^25^,^26^. As shown in Fig. 6A,B, very little modification of Akt was detected by MeHg, whereas CREB readily underwent *S*-mercuration by MeHg in a concentration-dependent manner. Nonreducing sodium dodecyl sulfide-polyacrylamide gel electrophoresis (SDS-PAGE), followed by atomic absorption spectrometry (AAS) analyses revealed that the Hg content resulting from covalent modification wild-type of CREB by MeHg was significantly greater than that of the serine mutant of Cys286 (Fig. 6C), supporting that the site of covalent modification of CREB by MeHg is, at least partially, Cys286.

## Discussion

The present study indicates that activation of the Akt/CREB/Bcl-2 signal transduction pathway in response to MeHg in SH-SY5Y cells does not increase linearly but rather follows a bell-shaped curve. These two distinct interactions of MeHg on the signal transduction pathways were related to the concentrations examined. For example, lower concentrations of MeHg (up to approximately 2 μM) significantly modified PTEN, but not Akt (Fig. 6A,B), and activated Akt so that it is able to phosphorylate CREB which then up-regulates Bcl-2 through transactivation of CRE (Figs 1 and 2). Using specific inhibitors for PI3K, we concluded the activation of Akt/CREB/Bcl-2 was mediated by MeHg (Figs 2 and 4). Pretreatments with wortmannin and Bcl-2 siRNA significantly enhanced MeHg-mediated cell death (Fig. 4), suggesting that while MeHg is found to cause apoptosis^12^,^13^,^14^, MeHg-dependent activation of Akt/CREB/Bcl-2 signalling itself plays an important role in protection against apoptosis at lower concentrations. Interestingly, irradiation-mediated caspase 3/7 activation was significantly inhibited by MeHg at a lower concentration (0.5 μM, 6 h), but not at higher concentrations (more than 2 μM), when it was significantly blocked by pretreatment with wortmannin (see Supplementary Fig. S3). Thus, it seems likely that this MeHg-induced hormetic effect that acts to protect irradiation-dependent cytotoxicity results from activation of Akt/CREB/Bcl-2 signaling during 0.5 μM MeHg exposure.

In contrast, MeHg at more than 2 μM diminished phosphorylation of CREB, thereby down-regulating Bcl-2 through blockage of CRE transactivation (Fig. 5). While CREB and Akt have 3 cysteine residues^28^ and 7 cysteine residues^29^, respectively, results from the BPM-precipitation assay indicated that CREB is more susceptible to MeHg than Akt in terms of *S*-mercuration mediated by MeHg (Fig. 6A,B). Previously, our matrix-assisted laser desorption and ionization time-of-flight mass spectrometry analysis revealed the environmental electrophile 1,2-NQ is covalently bound to CREB through Cys286, Lys290 and Lys319 and that *S*-arylation of CREB through Cys286 is essential for the blockage of DNA binding activity^27^, leading to a decreased level of Bcl-2^30^. These observations suggested that the down-regulation of CREB during exposure of SH-SY5Y cells to MeHg at more than 5 μM results from *S*-mercuration of this transcription factor at Cys286. Consistent with this notion, mutation of the cysteine to serine diminished covalent modification of CREB by MeHg (Fig. 6C). Overall, we speculate that covalent modification of CREB by environmental electrophiles such as 1,2-NQ and MeHg through Cys286 disrupts its DNA binding activity. Three cysteines are located in the basic leucine zipper domain of CREB, which is crucial for its binding to CRE as a dimer^31^,^32^. Thus, *S*-mercuration of CREB through Cys286 may change its conformation leading to disruption of the DNA binding involved in substantial apoptosis in SY-SY5Y cells.

Akt is well known to be activated by inactivation of PTEN by reactive oxygen species or carbonyl compounds^33^,^34^,^35^. Endogenous electrophiles such as Δ12-prostaglandin J_2_ and 4-hydroxynonenal can activate Akt through modification of cysteine residues in PTEN^36^,^37^,^38^. Similarly, MeHg was able to modify cellular PTEN in SH-SY5Y cells (Fig. 6A) and also inhibited enzyme activity of recombinant PTEN (see Supplementary Fig. S2). This indicated that *S*-mercuration of MeHg, resulting in inhibition of PTEN contributes, at least partially, to the activation of Akt during exposure to lower concentrations of MeHg. We have identified other molecular targets (e.g., Mn-SOD, nNOS, arginase I, SDH, Keap1 and UCH-L1) of MeHg using cell-free systems incubated with MeHg, cultured cells exposed to MeHg and experimental animals treated MeHg^5^,^6^,^7^,^8^,^9^. Among them, UCH-L1 is a cellular protein candidate that may be associated with activation of Akt^39^,^40^,^41^. Further studies are required to identify sensor protein(s) that easily undergo *S*-mercuration by MeHg, thereby activating Akt.

Several lines of evidence have indicated that there are multiple possibilities for activation of Nrf2/antioxidant responsive element pathway^42^. We previously reported that *S*-mercuration of Keap1 protein, the negative regulator of Nrf2, play a role in the substantial activation of Nrf2 in SH-SY5Y cells^43^,^44^. In the present examination, MeHg phosphorylated not only Akt but also GSK-3β during activation of Nrf2 mediated by MeHg in SH-SY5Y cells (Supplementary Fig. S1), suggesting that such a increased phosphorylation of GSK-3β may be, at least in part, involved in the Nrf2 activation caused by MeHg because GSK-3, which is inhibited by Akt, phosphorylates Ser residues in Neh6 domain of Nrf2 to form DpSGIpS motif-containing phosphodegron that is recognized by the β-transducin repeat-containing protein (β-TrCP)/Cul1-based E3 ubiquitin ligase complex (SCF), leading to degradation of Nrf2^45^. In addition, Nrf2 is found to regulate Bcl-2^47^, suggesting partial contribution of Nrf2 to up-regulation of Bcl-2 mediated by MeHg as well. Collectively, it is postulated that there is crosstalk for adaptive responses such as Akt and Nrf2 pathways induced by low-dose MeHg in SH-SY5Y cells.

**Figure 7:**
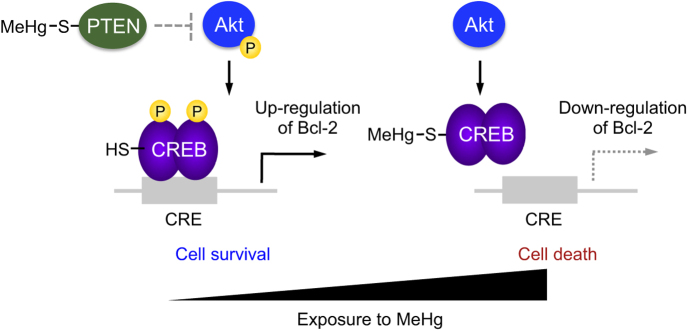
Biphasic regulation of Akt/CREB/Bcl-2 signaling mediated by MeHg.

Paracelsus (1493–1541), who was a scientist in addition to being doctor, introduced the concept that “poison is in everything… it is the dosage that makes something either a poison or a remedy”^48^. In other words, almost all chemicals have dual properties. The present study reports for the first time that MeHg causes a concentration specific activation (cell survival) and disruption (apoptosis) of the Akt/CREB/Bcl-2 signal transduction pathway in SH-SY5Y cells (Fig. 7). Although MeHg may also activate some apoptosis signal transduction pathways^12^,^13^,^14^, we postulated that the concentration-dependent *S*-mercuration of CREB, but not Akt, acts as a transducer of the MeHg effect, thereby repressing gene expression of the anti-apoptotic protein Bcl-2. We also speculate that activation of Akt/CREB/Bcl-2 signaling during exposure to MeHg at lower concentrations is, at least in part, involved in suppression of a threshold for MeHg-mediated cytotoxicity because blockage of Akt/CREB/Bcl-2 activation mediated by MeHg lowered the threshold for cytotoxicity.

## Methods

### Materials and Methods

MeHg and avidin-agarose was obtained from Sigma-Aldrich (St. Louis, MO, USA). *Escherichia coli* BL21 cells and trypsin were purchased from Promega Co. (Madison, WI, USA). Anti-GAPDH antibody and anti-Lamin B1 antibody were obtained from Santa Cruz Biotechnology Inc. (Santa Cruz, CA, USA). Anti-G6PD antibody, BPM, 4,6-diamidino-2-phenylindole (DAPI), annexin V fluorescein isothiocyanate (FITC), and propidium iodide were obtained from Bethyl Laboratories (Montgomery, TX, USA), Dojindo (Kumamoto, Japan), Nacalai Tesque Inc. (Kyoto, Japan), MBL (Aichi, Japan), Wako Pure Chemical Industries (Osaka, Japan), respectively. Anti-Akt, anti-CREB, anti-phosphorylated Akt (Ser473), anti-phosphorylated CREB (Ser133), anti-Bcl-2, horseradish peroxidase (HRP)-conjugated anti-rabbit antibodies, LY294002, and wortmannin were purchased from Cell Signaling Technology (Beverly, MA, USA). Alexa 488-secondary antibody, rhodamine phalloidin and Lipofectamine 2000 were obtained from Thermo Fisher Scientific (MA, USA). All other reagents used were of the highest purity available.

### Cells and cell culture

Human neuroblastoma SH-SY5Y cells were cultured in Dulbecco’s modified Eagle’s medium/Ham’s nutrient mixture F-12 with 10% fetal bovine serum, 2 mM L-alanyl-L-glutamine, and antibiotics (100 U/mL penicillin, 100 μg/mL streptomycin). Cultured cells were seeded at a density of 1 × 10^5^ cells/cm^2^ on a culture plate and maintained at 37 °C in a humidified incubator under an atmosphere of 5% CO_2_ and 95% ambient air. Before treatment, cells were cultured in serum-free medium overnight and then exposed to MeHg in serum-free medium to avoid MeHg binding to serum components.

### Western blot analysis

The cells were exposed to MeHg and washed with Dulbecco’s phosphate-buffered saline (D-PBS), then collected into 2% SDS and heated at 95 °C for 20 min. The protein concentrations were measured using a BCA Protein Assay Kit (Piece, Rockford, IL, USA). Each sample was mixed with SDS-PAGE loading buffer [62.5 mM Tris-HCl (pH 6.8), 8% glycerol (v/v), 2% SDS (w/v), 0.005% bromophenol blue (w/v) and 5% 2-mercaptoethanol (v/v)] and heated at 95 °C for 5 min. Proteins in the samples were separated by SDS-PAGE^49^ and then were electro-transferred on to polyvinylidene difluoride (PVDF) membranes (Bio-Rad Laboratories, Hercules, CA, USA) at 2 mA/cm^2^ for 1 h, according to the method of Kyhse-Anderson^50^. After blocking with 5% skim milk, the membranes were incubated with primary antibodies and then incubated with secondary antibodies coupled to HRP. Immunoreactive proteins were detected with a chemiluminescence system (Chemi-Lumi One L, Nacalai Tesque Inc.) and exposed to X-ray film (Konica Minolta Health Care Co., Tokyo, Japan). The bands were quantified using ImageJ software.

### Nuclear and cytoplasmic extraction

After exposure to MeHg, the cells were washed twice with D-PBS. Then, nuclear and cytoplasmic extracts were obtained by using NE-PER Nuclear and Cytoplasmic Extraction Reagents Kit (Thermo Fisher Scientific) under the manufacturer’s instructions. Protein concentrations were determined by a BCA Protein Assay Kit (Piece). Each samples was loaded on a SDS-PAGE and detected by Western blot analysis as described above.

### Purification of recombinant human UCH-L1 and CREB

Recombinant human CREB was purified as described previously^27^,^30^.

### Thermal decomposition gold amalgamation atomic absorption spectrophotometry analysis

Mercury concentrations in the gels were analysed by thermal decomposition gold amalgamation atomic absorption spectrophotometry (Mercury analyzer MA-3000; Nippon Instruments Corporation, Tokyo, Japan). Briefly, CREB recombinant protein in the gel was cut out using a gel band cutter (Nippon Genetics, Tokyo, Japan). The obtained bands (6 × 3 × 1 mm, W × H × D) were heated at 180 °C for 120 sec and 850 °C for 120 sec to vaporize the mercury. The mercury content in the gels was corrected for the protein amount.

### BPM-precipitation assay

The BPM assay was performed as described previously^25^. SH-SY5Y cells were seeded on a 60-mm dish and exposed to MeHg for 3 h. The cells were washed twice with D-PBS and then harvested with 1 mL of D-PBS. After centrifugation (5,000 × g for 5 min at 4 °C), the cells were lysed on ice for 20 min with 100 μL of radio-immunoprecipitation assay (RIPA) buffer (50 mM Tris-HCl, pH 8.0; 150 mM NaCl; 0.1% SDS; 0.5% deoxycholic acid; 1% NP-40) supplemented with protease inhibitors. The samples were incubated with BPM (20 μM) for 30 min at 37 °C. The reaction mixtures were centrifuged at 15,000 × g for 5 min at 4 °C and the supernatants were collected. BPM modified proteins were separated by addition of 50 μL of avidin-agarose suspended in RIPA buffer and further incubated at 4 °C overnight. After centrifugation (15,000 × g for 5 min at 4 °C), the precipitated agarose beads were collected and washed with 1 mL of RIPA buffer twice. The beads were heated with 20 μL of SDS-PAGE loading buffer at 95 °C for 20 min. After centrifugation at 5,000 × g for 5 min, the supernatants were subjected to SDS-PAGE and proteins were detected by western blotting as described above.

### Knockdown of Bcl-2

The siRNA for human Bcl-2–1 (sense; 5′-AUGCGGCCUCUGUUUGAUUtt-3′, antisense; 5′-AAUCAAACAGAGGCCGCAUtt-3′), Bcl-2–2 (sense; 5′-GAGAUAGUGAUGAAGUACAtt-3′, antisense; 5′-UGUACUUCAUCACUAUCUCtt-3′) and control siRNA (sense; 5′-UUCUCCGAACGUGUCACGUtt-3′, antisense; 5′-ACGUGACACGUUCGGAGAAtt-3′) were transfected with Hiperfect according to the manufacturer’s instructions (Qiagen).

### Immunohistochemistry

SH-SY5Y cells were cultured on a polyethylenimine-coated glass coverslips. After the exposure, the cells were washed with PBS, and fixed in 4% paraformaldehyde in PBS for 20 min. After the cells were washed with PBS and incubated with a blocking solution (2% BSA, 2% normal goat serum, and 0.4% Triton X-100 in PBS), phosphorylated Akt was labelled with the anti-phosphorylated Akt antibody (1:1000), and the proteins were visualized with Alexa 488-secondary antibody (1:1000). F-actin and the nucleus were also detected by rhodamine phalloidin (1:1000) and DAPI (0.1 μg/mL). Then, the cells were washed with PBS, mounted in fluorescent mounting medium (Dako Carpinteria, CA, USA) and observed using confocal laser scanning microscopy (ECLIPSE Ti, Nikon, Tokyo, Japan).

### Luciferase assay

DNA transfections were performed using Lipofectamine 2000 according to the manufacturer’s instructions. Briefly, cells were cultured in 12 well plates. Two micrograms of CRE-luciferase expression vector and an internal control vector containing Renilla luciferase (pRLTK, 0.2 μg) were mixed with Lipofectamine 2000 to allow formation of the plasmid DNA-lipid complexes. After transfection, the cells were treated with MeHg, and the luciferase activity in the cellular extracts was measured by the Dual Luciferase Reporter Assay System (Promega) according to the manufacturer’s instructions.

### Detection of apoptosis

SH-SY5Y cells were cultured in collagen-coated 8-well chamber plates. After pretreatment with or without 5 μM of wortmannin for 30 min, the cells were exposed to 1 μM of MeHg for 6 h. After washing with PBS, cells were incubated with annexin V FITC and propidium iodide in incubation buffer (10 mM HEPES [pH 7.4], 140 mM NaCl, 5 mM CaCl_2_) for 15 min. After washing with PBS, fluorescent images were captured using confocal laser scanning microscopy (ECLIPSE Ti, Nikon, Tokyo, Japan).

### Cell viability

The 3-(4,5-dimethylthiazol-2-yl)-2,5-triphenyltetrazolium bromide (MTT) assay was used to estimate cell viability. The MTT assay was performed as described previously^44^.

### Statistical analysis

The band intensities were quantified by ImageJ software, version 1.37. Statistical significance was assessed by *t*-test (Fig. 6C) or two-way ANOVA followed by a Bonferroni posttest. All *p* values are two tailed.

## Additional Information

**How to cite this article**: Unoki, T. *et al*. Methylmercury, an environmental electrophile capable of activation and disruption of the Akt/CREB/Bcl-2 signal transduction pathway in SH-SY5Y cells. *Sci. Rep.* **6**, 28944; doi: 10.1038/srep28944 (2016).

## Supplementary Material

Supplementary Information
