# Supplementary material for: Methylmercury, an environmental electrophile capable of activation and disruption of the Akt/CREB/Bcl-2 signal transduction pathway in SH-SY5Y cells
Source: Sci Rep. 2016 Jun 30;6:28944. doi: 10.1038/srep28944 (PMC4928048; doi:10.1038/srep28944)
Supplement: Supplementary Information [file srep28944-s1.pdf]

Methylmercury, an environmental electrophile capable of activation and disruption of the Akt/CREB/Bcl-2 signal transduction pathway in SH-SY5Y cells

Takamitsu Unoki[1, $], Yumi Abiko[1, $], Takashi Toyama[1, #], Takashi Uehara[2], Koji Tsuboi[1], Motohiro Nishida[3,4], Toshiyuki Kaji[5], Yoshito Kumagai[1,*]

[1]Faculty of Medicine, University of Tsukuba, Tsukuba, Ibaraki 305-8575, Japan

[2]Department of Medicinal Pharmacology, Graduate School of Medicine, Dentistry, and Pharmaceutical Sciences, Okayama University, Okayama 700-8530, Japan

[3]Division of Cardiocirculatory Signaling, Okazaki Institute for Integrative Bioscience (National Institute for Physiological Sciences), National Institutes of Natural Sciences, Okazaki, Aichi 444-8787, Japan

[4]Department of Translational Pharmaceutical Sciences, Graduate School of Pharmaceutical Sciences, Kyushu University, Fukuoka 812-8582, Japan

[5]Faculty of Pharmaceutical Sciences, Tokyo University of Science, Noda, Chiba 275-8510, Japan

[$] Equal contribution to the study

#Present address: Laboratory of Molecular and Biochemical Toxicology, Graduate School of Pharmaceutical Sciences, Tohoku University

[*]Address correspondence to Yoshito Kumagai, Faculty of Medicine, University of Tsukuba, 1-1-1 Tennodai, Tsukuba, Ibaraki 305-8575, Japan; Tel & Fax, +81-29-853-3133; E-mail, yk-em-tu@md.tsukuba.ac.jp

Table of contents

Supplementary Materials and Methods. ……………………………………………………2

Supplementary Figure S1. ………………………………………………………………3

Supplementary Figure S2. ………………………………………………………………4

Supplementary Figure S3. ………………………………………………………………5

Supplementary Reference. ………………………………………………………………6

Antibodies

Anti-GSK-3β and anti-phosphorylated GSK-3β (Ser9) were obtained from Cell Signaling Technology (Beverly, MA, USA). Anti-Nrf2 antibody was obtained from Santa Cruz Biotechnology Inc. (Santa Cruz, CA, USA).

PTEN activity

PTEN activity was determined as previously reported[1]. Recombinant GST-PTEN was purified and the protein (0.15 μM) was assayed for lipid phosphatase activity against phosphatidylinositol 3,4,5-triphosphate (PI(3,4,5)P_3). Briefly, recombinant PTEN protein was incubated with or without MeHg for 1 h at 37 °C in reaction buffer (10 mM HEPES, pH 7.2 and 150 mM NaCl) and then incubated with 50 μM dioctanoyl PI(3,4,5)P_3 for 10 min at 37 °C. The reaction was stopped by adding 50 mM *N*-ethylmaleimide and the samples were centrifuged at 20,000×g for 20 min at 4 °C. Phosphate released into the supernatant was detected colorimetrically with malachite green reagent.

Cell viability

A lactate dehydrogenase (LDH) assay was used to estimate cell viability. SH-SY5Y cells were seeded in a 25 cm^2 flask. After exposure of the cells to X-rays, the conditioned medium was harvested, and an aliquot was used for the assay of LDH activity. The LDH leakage assay was performed using the CytoTox Non-radioactive Cytotoxicity Assay kit (Promega) according to the manufacturer's instructions.

Caspase activity

Caspase 3/7 activity was measured using the Caspase-Glo 3/7 assay kit (Promega) according to the manufacturer's instructions. Briefly, cells were seeded in a 25 cm^2 flask. After X-ray radiation, the cells were lysed in cell lysis buffer (20 mM Tris-HCl, pH 7.5; 150 mM NaCl; 2.5 mM sodium pyrophosphate; 1 mM EDTA; 1 mM sodium orthovanadate; and 1% Triton-X). The cells were then centrifuged, and the supernatant was collected. Each sample was normalized to a protein content of 30 μg, and then mixed with Caspase-Glo 3/7 reagent for 3 min at room temperature. The luciferase activity was measured by luminometer (TD20/20, Turner Designs, Sunnyvale, CA, USA).

Supplementary Figure S1.

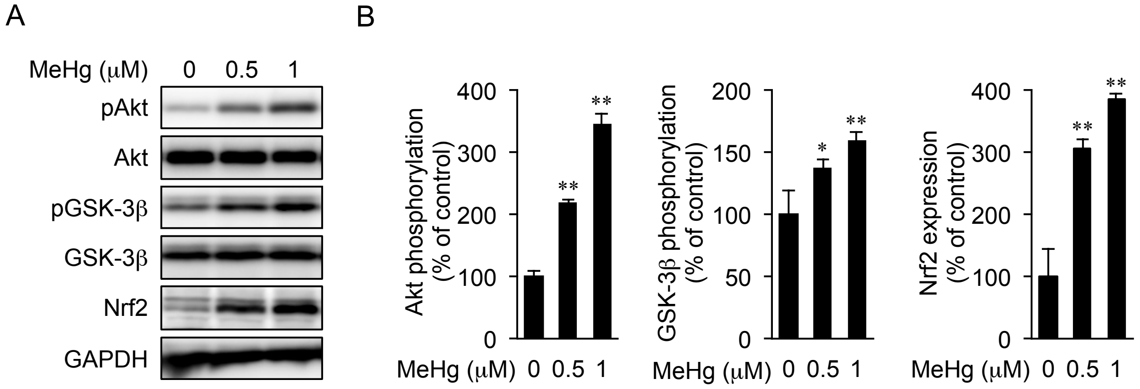

Supplementary Figure S1. Activation of Nrf2 and phosphorylation of Akt and GSK-3β during exposure of SH-SY5Y cells to MeHg. SH-SY5Y cells were treated with indicated concentration of MeHg for 12 h. The whole cell lysates were subjected to western blotting and the band intensities were quantified. (A). The ratio of phosphorylated/total Akt and GSK-3β intensity, and Nrf2 intensity in control were defined as 100%. *$p < 0.05$ and **$p < 0.01$ *vs*. control. Each value is the mean ± S.D. of three determinations (B).

Supplementary Figure S2.

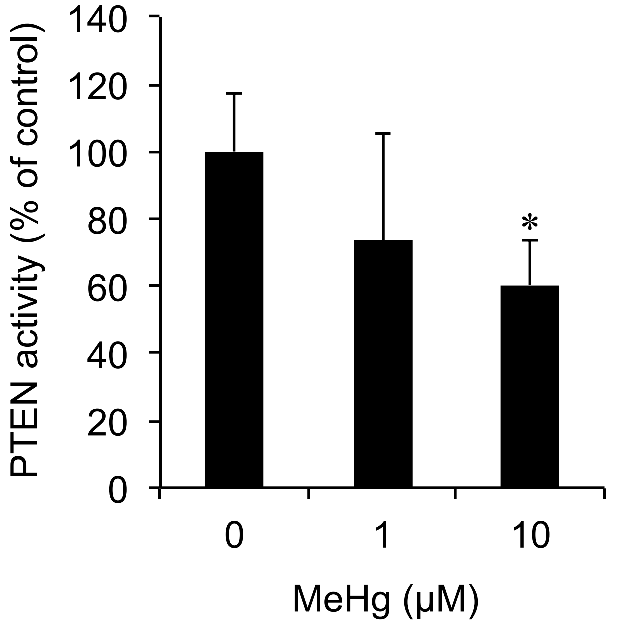

Supplementary Figure S2. Inhibition of PTEN activity by MeHg. Recombinant PTEN (0.15 μM) was incubated with MeHg for 1 h. PTEN activity was measured as described in the Supplementary Methods section. *$p < 0.05$ *vs*. control. Each value is the mean ± S.D. of three determinations.

Supplementary Figure S3.

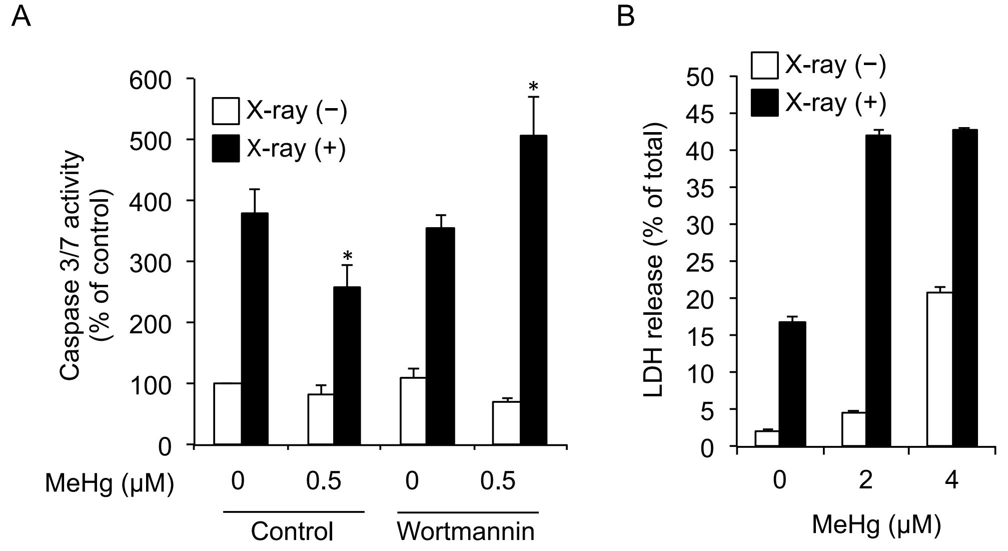

Supplementary Figure S3. Suppression of Xr-ray-dependent cytotoxicity by Akt activation mediated by MeHg. SH-SY5Y cells were pretreated with 0.5 μM of wortmannin for 30 min prior to MeHg treatment for 6 h, and then exposed to X-rays for 6 h (6 Gy). The caspase activity in the cells was measured as described in the Supplementary Methods section (A). SH-SY5Y cells were treated with MeHg for 6 h, followed by X-rays irradiation for 6 h (6 Gy). The LDH release from the cells to the extracellular space was measured as described in the Supplementary Methods section (B). *$p < 0.05$ *vs*. control. Each value is the mean ± S.E. of three determinations.

Supplementary Reference

1. Numajiri, N. et al. On–off system for PI3-kinase–Akt signaling through S-nitrosylation of phosphatase with sequence homology to tensin (PTEN). *Proc. Natl. Acad. Sci. USA* **108**, 10349–10354, (2011).
